# Understanding Behçet’s Disease in the Context of Innate Immunity Activation

**DOI:** 10.3389/fimmu.2020.586558

**Published:** 2020-10-20

**Authors:** Sandro F. Perazzio, Luis E. C. Andrade, Alexandre W. S. de Souza

**Affiliations:** Division of Rheumatology, Universidade Federal de São Paulo, São Paulo, Brazil

**Keywords:** Behçet’s disease, innate immunity, phagocytes, neutrophil hyperactivity, alarmin, *Streptococcus sanguinis*

## Abstract

Behçet´s disease (BD) is a heterogeneous condition consisting of idiopathic systemic vasculitis affecting large and small blood vessels of different types (i.e., arteries, veins, or capillaries). The disease frequently occurs in young adults without gender predilection, differently from several other autoimmune conditions. This challenging illness has recently been proposed by some authors as an example of complex autoinflammatory syndrome. Although much remains unanswered about BD pathogenesis, recent understanding of some aspects of innate immunity have clarified a few issues (and raised others). HLA-B*51 represents the strongest genetic risk factor for BD to date, albeit several other HLA-independent loci have also been associated with the disease. The consistent hyper-reactivity against *Streptococcus sanguinis* antigens and alterations in oral and gut microbioma suggests that infectious agents may play an important role. Moreover, functional abnormalities of pattern recognition receptors, especially Toll-like receptors in monocytes, have been demonstrated in patients with BD and can be associated with the development of the disease. Neutrophil hyperactivity is one of the most consistent findings in BD pathogenesis, as demonstrated by exacerbated constitutive oxidative burst, chemotaxis and NET formation. However, some studies suggest that the phagocyte-activated status in BD is not primary to the disease itself, but rather restricted to a fraction of patients with severe disease activity, and probably secondary to activating soluble factors carried by serum/plasma from BD patients. Herein we review the state of the art on BD etiopathogenesis with special emphasis on the participation of the innate immune system

## Introduction

Behçet’s disease (BD) is an idiopathic systemic vasculitis affecting large and small blood vessels. It was initially described as recurrent oral and genital ulcers associated with anterior uveitis with hypopyon by Hulusi Behçet (1) and Benediktos Adamantiades (2) in the 1930s. Progressively, however, a variety of musculoskeletal, neurological, gastrointestinal and vascular manifestations was associated to the syndrome.

The epidemiology of BD exhibits an interesting geographic distribution throughout the ancient “Silk route”, with higher prevalence in Turkey, Iran and Japan. The disease frequently occurs in young adults (mean age: 25–30 years old) (3). The lack of gender predominance is one of the arguments favoring the non-autoimmune nature of BD pathogenesis. This challenging illness received special attention in the last years, culminating with novel insights on the possible autoinflammatory etiopathogenesis of the disease, which encouraged some authors to consider BD as an example of complex autoinflammatory syndrome (4). Much remains unanswered in BD pathogenesis, as recent progress in the understanding of some aspects of the innate immunity has raised unforeseen questions. Herein we review the state of the art on BD etiopathogenesis with special emphasis on the participation of innate immunity activation.

## The Immunogenetic Basis of Behçet’s Disease

### Histocompatibility Leucocyte Antigen

Histocompatibility leucocyte antigen (HLA)-B*51 represents the strongest genetic risk factor for BD to date. It was initially reported in the Japanese (5) and reproduced among several other ethnic groups (6). A meta-analysis reported an overall odds ratio of 5.78 (95% CI=5.00–6.67) for HLA-B*51 carriers to develop BD, independently of the ethnicity (6). Similar results were confirmed in two different genome-wide association studies (GWAS) in Japanese (7) and Turkish BD patients (8). However, recently other loci have been demonstrated to increase the risk of BD as well. Kuranov et al. (9) studied HLA-B*51-negative patients and showed a significant association of BD and HLA-Bw4-80I, an epitope present on B locus-derived proteins, characterized by the presence of an isoleucine at amino-acid position 80 in the α1 helix of the HLA-B*04. Additionally, these authors found an association with HLA-A*26 independent from HLA-B*51, which was confirmed in other studies (8, 10). In addition, Hughes et al. (10) demonstrated that HLA-A*03, B*15, B*27, B*49 and B*57 also contribute to BD risk independently, although this has not been replicated to the moment. Another study identified additional independent risk factors for BD located at HLAB/MICA and at the region between HLA-F and HLA-A (11).

### Other Genetic Risk Factors

GWAS in BD patients identified extra-HLA genetic risk factors. By analyzing 311,459 SNPs in 1,215 BD patients and 1,278 controls, Remmers et al. (8) identified two novel susceptible loci for BD: *IL23R-IL12RB2* and *IL10* (allele rs1518111 A, associated with low mRNA and protein expression). Recently, association between *IL10* polymorphisms and BD was also demonstrated in Chinese patients (12). These data emphasize the possible role of IL-10 in BD pathogenesis and raise the question of possible participation of adaptive immunity, especially Th17 and Treg cells, in BD (13).

Copy number variation (CNV) of Complement component *C4* genes was investigated in BD. In contrast to systemic lupus erythematosus (14), there is an increased frequency of more than 2 copies of the *C4A* gene in BD patients and this represents a risk factor independent from HLA-B*51 (15). Moreover, the authors also demonstrated that BD patients with high *C4A* copy number had increased production of IL-6, an important mediator of the innate immunity acting as an acute phase reactant.

Another interesting observation is the presence of a specific chromosomal abnormality in a number of patients with BD: trisomy of chromosome 8 (16, 17). Considered a risk factor for myeloid leukemia (18) and myelodysplastic syndrome (19), appearing in 5-10% of patients, trisomy 8 also seems to play a role in BD. As shown in a recent study, its frequency was reported as high as 86% in patients with concurrent BD and myelodysplastic syndrome (20). Apparently, these cases present frequently with prominent gastrointestinal involvement and no geographical preference (21). Interestingly, chromosome 8 harbors some pivotal genes related to innate immunity modulation and NF-κB pathway activation, such as *IKBKB*.

## Alarmins and Microorganisms

Alarmins are a group of proteins with the ability of initiating the innate immune response after quick release following cell necrosis. Alarmins activate pattern recognition receptors (PRR), such as Toll-like receptors (TLR), and are essential to restore homeostasis after tissue damage. In fact, alarmins are considered a subtype of DAMP (damage-associated molecular patterns), which consist of stereotyped molecular patterns shared by molecules originated after exposure to physical or chemical agents capable of inducing tissue damage (e.g., radiation, heat and cold, among others).

The High Mobility Group Box 1 (HMGB1) is probably the most studied alarmin in systemic autoimmune rheumatic diseases (22–26). Ahn et al. (27) demonstrated that HMGB1 serum levels are increased in BD patients, especially those with gastrointestinal involvement. Conversely, our group found higher HMGB1 levels in BD patients compared to controls, regardless of disease activity, disease manifestations or therapy with prednisone and azathioprine (28). Han et al. (29) reported increased serum levels of alarmin S100A12 in BD, independently of disease activity, although at higher magnitude in active phase. Accordingly, S100A12 serum levels decreased after the treatment and the protein expression was increased in skin biopsies of active erythema nodosum lesions from BD patients.

There is also some evidence regarding the role of microorganisms on BD. The hyper-reactivity against *Streptococcus sanguinis* antigens and the homology and potential cross-reactivity of some of its proteins with human heat-shock proteins (HSP) (30), as exemplified by the activation of T γδ^+^ cells by the pathogen and HSP 60/65 kDa (31), suggest this infectious agent might play an important role in BD pathogenesis (32). Herpes simplex virus 1 (HSV-1), *Staphylococcus aureus*, *Mycobacterium tuberculosis* and some *Prevotella* species have also been identified as potential candidates (33). HSV-1 RNA and DNA were found in increased frequency in cells from BD patients (34, 35). Moreover, mycobacterial HSP peptides stimulate γδ^+^ T cells from BD patients, which, in turn, are increased in peripheral blood and mucosal lesions (36). Despite these intriguing set of data, a direct causal relationship between infectious agents and BD, as well as the precise role of γδ^+^ T cells on the disease pathogenesis, remain unclear.

## Toll-Like Receptors and Other PRR

Evidence of increased serum levels of alarmins and hyperactivity against some microorganisms turns plausible the hypothesis that PRR participate in BD pathogenesis. Indeed, functional abnormalities of PRR and their activation cascades have been identified in previous studies and can be associated with BD development. Yavuz et al. (37) demonstrated that TLR6 expression is significantly increased in granulocytes from BD patients after stimulus with *Streptococcus sanguinis* or HSP-60 compared to rheumatoid arthritis patients and healthy controls. Interestingly, monocytes from BD patients presented lower TLR2 expression after the same stimuli. Of interest, Neves et al. (38) and Do et al. (39) showed that TLR2 and TLR4 expression in monocytes from BD patients was constitutively increased; however, this finding was not observed in neutrophils from these patients.

A recent GWAS with 2,461 BD cases and 2,458 healthy controls showed protective *TLR4* and *NOD2* polymorphisms, respectively associated with decreased response to lipopolysaccharide and muramyl dipeptide (40). Furthermore, a multicenter study with Chinese and Dutch patients has provided evidence that polymorphisms in TLR2 are involved in ocular BD susceptibility (41). A SNP of *TIRAP*, a MyD88-adapter-like molecule with a regulatory role in TLR2 and TLR4 signaling, has been associated with BD in a British cohort (42), but results were not replicated in Middle-Eastern, Turkish or Italian patients (43).

Taken together, these findings implicate innate immunity and bacterial sensing mechanisms as important players in BD pathogenesis, with participation of diverse gene polymorphisms according to different ethnicities, and represent a promising investigational area in BD.

## The NF-κB Pathway

Downstream signaling leading to internalization of the two nuclear factor κB subunits (p50 and p65) represents the canonical signal transduction pathway after activation of several PRR. Despite the inflammatory characteristics of BD suggesting NF-κB hyperactivation in BD patients (44), there are few studies in the area, but this has progressively been changing lately.

Polymorphisms in *NFKB1* promoter (–94 insertion⁄deletion ATTG) (45) and *NFKBIA* (rs696) (46) were demonstrated to enhance the risk for BD in the Turkish population. It seems that NF-κB plays a pivotal role in controlling T cells apoptosis in BD. Although CD95 is highly expressed on T cells from BD patients, Todaro et al. (47) demonstrated decreased sensitivity to CD95-induced apoptosis, possibly attributed to the inhibitory action of anti-apoptotic genes (*CFLAR*, *BCL2L1*, *BCL2*, *CASP3*, *CASP8*) and up-regulated expression of Iκκ, IκB, and NF-κB. Interestingly, thalidomide, a therapeutic agent used in severe mucocutaneous manifestations of BD, and NF-κB small interfering RNA down-regulated cFLIP and Bcl-xL expression levels, ultimately increasing activated T cells sensitivity to CD95-induced apoptosis in BD.

Constitutive NF-κB canonical pathway hyperactivation in BD phagocytes was previously reported by our group, as indicated by the over-expression of phosphorylated p65 subunit (44). Similarly, a monogenic form of an autoinflammatory disorder resembling BD was described in five families carrying heterozygous germline mutations of *TNFAIP3*, a potent inhibitor of the NF-κB canonical pathway (48). The mutant *TNFAIP3*-derived transcript A20 is not capable of modulating intracellular signaling, ultimately culminating in phagocyte hyperactivation and increased NF-κB-mediated proinflammatory cytokines secretion. Moreover, carriers of *NFKB1* variants have been reported to present a monogenic BD-like disease, characterized by pathergy-like lesions and striking macrophage inflammasome activation. Finally, an autosomal-dominant mucocutaneous ulceration disorder was recently associated with *RELA* mutations, encoding the NF-κB subunit p65 (49).

Previous studies further support the clinical overlap between BD and other autoinflammatory diseases with shared etiopathogenesis, such as familial Mediterranean fever (4, 50). *MEFV* M694V mutation frequency is increased in Turkish BD patients (40). Rare genetic variants of undetermined significance in inflammasome components upstream of NF-κB have also been found in BD patients, especially *NOD2* and *NLRP3* (51–53). Some of these variants may contribute to the disease onset, but others could be only single nucleotide polymorphisms without any effect. Anyhow, inflammasome-activated NF-κβ pathway dysregulation seems to be a common finding in disorders with BD-like phenotypes (54).

## Endothelial Cell Dysfunction

As a pro-thrombotic condition, one would expect the existence of some sort of endothelial dysfunction in BD. A study in Turkish patients showed that patients with active disease presented lower nitric oxide serum levels than those in remission (55). Since endothelial cells are major producers of nitric oxide, the authors suggested a putative dysfunction in these cells. This would be probably mediated by increase in oxidative stress due to augmented malondialdehyde (a metabolite of polyunsaturated lipids oxidation by reactive oxygen species – ROS) serum levels in active BD patients.

Fadini et al. (56) originally demonstrated a progressive decrease of circulating endothelial progenitor cells in BD patients, which might represent a vascular damage mechanism, since these cells are involved in vascular homeostasis and repair. The authors also showed a positive correlation between the number of endothelial progenitor cells and both BD activity score and C-reactive protein.

A systematic review aimed to evaluate subclinical atherosclerosis in BD by endothelial-mediated dilatation and by measurement of intima media thickness (IMT) of carotid arteries (57). Among nine studies, endothelial-mediated dilation was demonstrated to be impaired in BD even in inactive state. IMT was greater in BD patients, despite considerable variation that reflects the clinical heterogeneity of the disease.

## Neutrophil Hyperactivity

Clinical and pathological data strongly suggest that neutrophil hyperactivity is a prominent feature in BD pathogenesis. Exacerbated neutrophil activity can be determined by evaluating oxidative burst, phagocytic and microbicide activities, activation of intracellular signaling pathway, among others. Takeno et al. (58) showed that ROS production is increased not only in BD patients but also in asymptomatic HLA-B*51 carriers and even in transgenic mice expressing HLA-B*51. These observations suggest a mechanistic connection between the already known immunogenetic background of BD and its pathogenesis.

Carletto et al. (59) described that peripheral blood and skin (obtained by cutaneous abrasion) neutrophils from patients with active BD present higher migration capacity than those from healthy controls and BD patients with inactive disease. The increased migration capability was normalized when patients attained remission, suggesting that this mechanism is involved in the inflammatory state of the disease. In contrast, no significant abnormalities were observed in other neutrophil functions, such as adhesion or superoxide production after zymosan, phorbol-myristate-acetate (PMA) or N-formylmethionine-leucyl-phenylalanine (fMLP) stimuli. However, Yoshida et al. (60), using a chemoluminescence method to determine the superoxide production in 20 BD patients and healthy controls, demonstrated a significantly higher superoxide production by neutrophils from BD patients after the same stimuli used by Carletto et al. (59).

Eksioglu-Demiralp et al. (61) studied the oxidative burst after stimuli with PMA or fMLP and the phagocytic activity against *E. coli* in neutrophils from healthy controls, BD patients (HLA-B*51 carriers or not), septic patients and patients with inflammatory arthropathy (namely, rheumatoid arthritis and ankylosing spondylitis). Oxidative burst was decreased in stimulated neutrophils from BD and septic patients, suggesting that phagocytes were exhausted and hypo-responsive *in vitro* due to previous *in vivo* hyper-activation. Interestingly, phagocytic activity was significantly increased in septic and inflammatory arthropathy groups, but did not differ between BD patients and healthy controls.

The same Turkish group published in 2002 another study reassessing phagocytic activity and oxidative burst profile after PMA stimulus of neutrophils from healthy controls, BD patients and “inflammatory patients” (septic, primary vasculitis, systemic lupus erythematous, osteomyelitis and pneumonia) (62). Exclusively BD patients presented a decreased oxidative burst after stimulus, which was inhibited by nitric oxide synthase inhibitors, although significantly less than the healthy controls. There was no difference in phagocytic activity among the groups. Once again, the authors attributed the results to a possible *in vivo* pre-activated/exhaustion state of BD neutrophils.

Altogether, these data support the concept that neutrophils play a pivotal role in BD pathogenesis. However, there might be other factors contributing to BD development, many of them still unknown. It is unclear, for example, if the striking neutrophil hyperactivation occurs constitutively or if it is secondary to a yet unknown stimulus, such as bacterial (e.g.: *Streptococcus*) or viral infections. Therefore, in an attempt to clarify this doubtful issue, our group designed a study aiming to assess the classical phagocyte functions (i.e., oxidative burst, *in vitro* cytokine production, phagocytic and microbicide activities) before and after stimulus with pathogens and several microbial components in 30 healthy controls, 25 septic patients, 31 inactive, and 30 active BD patients (63). We observed that phagocytes from BD patients with severe manifestations exhibit significantly higher oxidative burst activity, both before and after PMA stimulation, compared to cells from patients with mild BD manifestations. Furthermore, we found significant positive correlations between BD patients’ scores on the simplified *Behçet´s Disease Current Activity Form* (BR-BDCAF), a validated tool to measure disease activity, and *Streptococcus sanguinis*-stimulated production of IL-23 by peripheral blood mononuclear cells (PBMC) and IL-8 by neutrophils. In addition, significant positive correlations were also found between BR-BDCAF score and constitutive production of TNFα, IFNγ, IL-6, and IL-23 by PBMC. Thus, our study corroborates the participation of phagocyte in BD pathogenesis by the evidence that patients with severe BD exhibit phagocytic dysfunction and some extent of constitutive activation.

In contrast, an important aspect of neutrophil biology has largely been ignored despite the striking body of evidence of involvement of these cells in BD: neutrophil extracellular traps (NET) release. Our group originally showed an increased constitutive NET release in BD patients (64). Interestingly, NETosis was markedly stimulated by soluble CD40L, especially from plasma of active BD patients. Similarly, Le Joncour et al. (65) recently demonstrated that circulating NET components are elevated in active BD patients, mainly in those with vascular involvement, suggesting that NET may represent a potential therapeutic target for BD-associated thrombotic risk.

**Figure 1:**
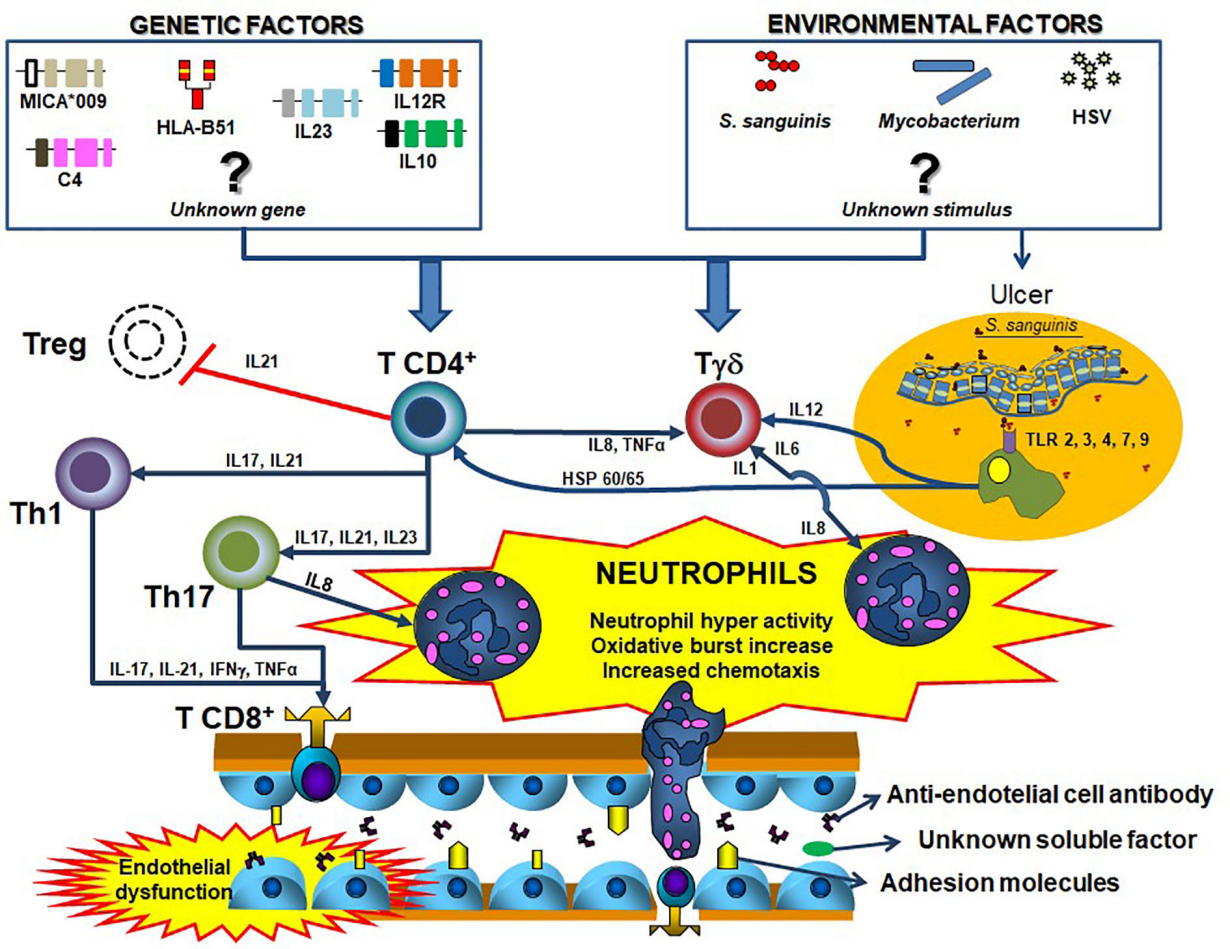
Summary of the possible Behçet´s disease pathogenesis. Distinct T helper cells mainly Th1 and Th17 have the ability of stimulating T effectors and T regulatory cells especially by the action of cytokines. Although some remain unknown, several genetic (e.g.: HLA-B*51, MICA, C4 copy number variation, among others),,, and environmental factors (e.g., *Streptococcus sanguinis*, *Herpes-simplex virus*, mycobacteria, among others) are involved in the process, by facilitating the activation of T cells. Similarly, the antigen presenting cells, especially macrophages from mucosa, stimulate immune cells by Toll-like receptors activation. All these innate and adaptive immune pathways culminate with the sequential neutrophil activation, considered the most important element in BD pathogenesis. blue closed arrows represent stimulation of a cell subtype mediated by cytokines, while red open lists represent inhibition.

Despite some controversy in the literature regarding neutrophil dysfunction in BD (summarized in **Table 1**), the bulk of evidence suggests that the activated status of phagocytes in BD is not a constitutive feature, but rather restricted to a fraction of severely active patients, and probably secondary to an unknown soluble factor (**Figure 1**).

**Table 1:** Controversies regarding phagocyte activity in Behçet’s disease (BD).

Author	Year	Brief description	Reference
Takeno et al	1995	Increased oxidative burst in BD patients and HLA-B51 healthy controls.	(58)
Sahin S et al	1996	Increased adhesion.	(66)
Carletto A et al	1997	Increased migration (active BD only). No difference regarding oxidative burst.	(59)
Yoshida et al	1998	Constitutively increased oxidative burst in BD neutrophils. Increased oxidative burst in neutrophils from healthy controls after pretreatment with serum from BD patients.	(60)
Eksioglu-Demiralp E et al	2001	Decreased oxidative burst. No difference regarding phagocytic activity compared to healthy controls.	(61)
Atalay G et al	2002	Decreased oxidative burst (active BD only). No difference regarding phagocytic activity.	(62)
Neves et al	2009	Both normal and BD neutrophils increased chemotactic capacity after incubation with BD plasma. No difference regarding chemotaxis.	(38)
Perazzio et al	2015	Increased oxidative burst (severe BD only). Positive correlation between activity score and constitutive or *Streptococcus sanguinis*-stimulated production of cytokines *in vitro*. No differences regarding phagocytic and microbicide activities.	(44)
Perazzio et al	2017	Plasma from BD patients exerted a stimulus on neutrophil extracellular traps release and oxidative burst, probably induced by sCD40L	(44)
Le Joncour et al	2019	Circulating neutrophil extracellular traps markers are elevated in BD and contribute to the procoagulant state	(65)

## Role of Monocytes in BD

Like neutrophils, monocytes are important agents of the innate immune system by means of their phagocytic activity, oxidative burst and cytokine production. Thus, it is reasonable to suppose that their function and response to stimuli may bear some similarity to those of neutrophils, especially in diseases with neutrophil hyperactivity.

Indeed, Gogus et al. (67) showed that monocytes from BD and familial Mediterranean fever patients present higher oxidative burst activity than those from rheumatoid arthritis patients and healthy controls, especially when stimulated by sodium monourate crystals. Moreover, interactions between neutrophils and monocytes have received particular attention. For example, peptides released from activated human neutrophils stimulate monocyte adhesion and transmigration as well as macrophage oxidative burst (68).

Interestingly, monocytes from BD patients present higher expression of TLR2. Moreover, bacterial-derived lipoteichoic acid activated TLR2 increases neutrophil chemotaxis and adhesion to endothelial cells (38). Furthermore, active DB is associated with higher expressions of TLR2 and TLR4 in monocytes, as well as with a higher frequency of pro-inflammatory CD14^+^CD16^+^ monocytes in the peripheral blood compared to healthy controls (39). *In vitro* lipopolysaccharide-stimulated monocytes from BD patients produced similar amounts of TNFα compared to healthy controls cells; however, a higher *in vitro* production of TNFα was observed in monocytes from clinically active BD patients in comparison to those from quiescent BD patients (69). It would be intriguing to further explore this aspect in BD since monocytes seem to play a role in its pathogenesis as these cells may contribute to neutrophil activation after bacterial triggers in BD patients.

## Soluble Factors and Autoantibodies

Cytokines and soluble receptors are major effectors of innate immunity and some of them have already been associated with BD pathogenesis. A previous study showed an increased expression of Th1 cytokines (IL-12, IL-18, and IFNγ) in skin and oral ulcers from active BD patients (70). In fact, Yanaginori et al. (71) demonstrated that stimulation with Streptococcal antigens specifically increased gene and protein expression of IL12p40, in conjunction with IL12p70 induction, in PBMC from BD patients. This finding provides evidence for Th1-skewed anti-bacterial host response mediated by IL-12 in BD patients. Two studies showed that serum and cerebrospinal fluid (CSF) levels of IL-15 and IL-18, two crucial Th1 cytokines, were increased in neuro-Behçet patients (72, 73).

Moreover, in the strict context of innate immunity, some studies showed that serum levels of IL-8, a potent neutrophil activator and chemotactic factor, are increased in BD patients (66, 74), especially in active disease (75). IL-8 levels were also elevated in synovial fluid from BD compared to osteoarthritis patients, which suggests an important role for this cytokine in BD pathogenesis (76). Inflammasome or NF-κB-derived IL-1β, TNFα, and IL-6 are also representatives of strictly innate immunity soluble mediators and act individually or altogether in systemic inflammation, inducing acute phase reactants and phagocyte activation. These cytokines may play pivotal role on BD etiopathogenesis, as therapeutical blockade is indicated by current clinical guidelines (77). Among immunobiological therapies for BD, certainly anti-TNF are the most frequently prescribed and are indicated for refractory mucocutaneous lesions, peripheral vascular symptoms (deep venous thrombosis and arterial aneurisms) and as alternative for parenchymal central nervous system and ocular manifestations. Anti-IL-6 therapy has been indicated on refractory central nervous system manifestations (78–80) and anti-IL-1β showed promising results for severe ocular (81, 82) and mucocutaneous clinical phenotype (83, 84).

Interestingly, Alpsoy et al. (85) demonstrated that *IL8* gene expression was increased in macrophages from BD patients and healthy controls after incubation with serum from active BD patients. Similarly, (85) several other studies demonstrated the capacity of serum or plasma from BD patients to stimulate the innate immune system. Yoshida et al. primed neutrophils from healthy controls with BD serum and observed an increase in the production of superoxide similar to that observed after stimuli with zymosan, PMA or fMLP. (60). However, the absence of objective BD disease activity determination was a caveat of that study. Sahin et al. (66) demonstrated an increased adhesion ability of normal neutrophils to human umbilical vascular endothelial cells and increased expression of adhesion molecules (CD11a, CD18, and ICAM-1) when exposed to BD serum, compared to the stimulus of normal serum. However, the authors could not find any difference between serum from patients with active and inactive disease, possibly due to similarly high IL-8 serum levels in both groups. Another study from the same group showed that BD patients presented higher monocyte expression of CD14, a monocyte-activating marker, as well as higher soluble CD14 serum levels than healthy controls (86). Furthermore, the supernatant of BD monocyte culture significantly increased the adhesion ability of normal neutrophils to endothelial cells *in vitro*. These results indicate that BD monocytes are active and produce a milieu of pro inflammatory cytokines, which may play a role in the chronic inflammation of BD.

Neves et al. showed that chemotaxis was similar in neutrophils from BD and normal controls after stimulation with lipoteichoic acid (38). Interestingly, both healthy and BD neutrophils presented increased chemotactic capacity when incubated in the presence of BD plasma or stimulated with C5a, B4 leukotriene or fMLP. Similarly to Sahin et al. (86), CD14 expression in monocytes and soluble CD14 serum levels were increased in BD patients. Additionally, the authors showed a positive correlation between BDCAF and soluble CD14 serum levels, suggesting that the soluble proinflammatory factors produced in BD correlate with disease activity.

Our group also demonstrated that NET release and oxidative burst were stimulated with plasma from BD patients (64). In addition, markedly elevated sCD40L serum levels in conjunction with CD40L overexpression on CD4^+^ T cells from BD patients were observed. Interestingly, we originally described that both NET release and oxidative burst were exacerbated by recombinant sCD40L and decreased after sCD40L blockade, suggesting a possible role of this mediator on BD pathogenesis.

Serum and plasma seem not to be the only carriers of soluble factors related to phagocyte activation in BD. Chemokine levels in aqueous humor are apparently also increased in BD patients with ocular manifestations. El-Asrar et al. (87) demonstrated that CXCL1 and CXCL10 were significantly higher in aqueous humor of patients with BD compared to patients with Vogt-Koyanagi-Harada disease and HLA-B*27-associated uveitis. Additionally, CCR5 and CXCR3 had increased expression in biopsy specimens of oral ulcers from BD patients compared to healthy controls (88), and MIP-1β (macrophage inflammatory protein 1β) had increased serum levels in BD (70), indicating a Th1-skewed immune response on BD immunopathology. Interestingly, another study showed increased expression of transmembrane CXCL16 on circulating plasmacytoid dendritic cells from BD patients, which might contribute to the high serum IFN-α levels seen in patients with BD (89).

Although BD is not a typical autoantibody-associated condition, anti-α-enolase antibodies have been associated with the disease. One study demonstrated that *Streptococcus sanguinis* or BD serum stimuli increase α-enolase expression on human microvascular endothelial dermal cells, a target for autoantibodies observed in a fraction of BD patients (90). Thus, hyper-expressed α-enolase could react to anti-α-enolase antibodies present in BD serum, eliciting immune response (91). Additionally, the same authors previously identified that heterogeneous nuclear ribonucleoprotein (hnRNP) A2/B1 is a cross-reactive target of anti-α-enolase antibodies and that *Streptococcus sanguinis* or serum from active BD patients are capable of inducing *in vitro* expression of hnRNP A2/B1 in human microvascular endothelial dermal cells (92). Orem et al. (93) also described that plasma from BD patients impaired nitric oxide production by human umbilical vascular endothelial cells, suggesting an inhibitory effect over endothelial NO synthase.

Despite several pieces of evidence, the literature is still controversial regarding the potential role played by a possible soluble phagocyte- or endothelial-activating factor carried by serum or plasma (**Figure 1**). As discussed above, it is unclear whether phagocyte activation is constitutive in BD or secondary to a soluble factor stimulation. Additionally, even considering the existence of such a factor, the identity and the cells responsible for its production remain unknown. Therefore, this is an area of great interest and further research is warranted to clarify these questions.

## Innate Versus Adaptive Immunity: A Paradigm From the Past

Although BD is considered an example of strong innate immunity activation, it is important to highlight that not all immune cells can be assigned strictly to either the innate or the adaptive arm of the immune system. The existence of bridge populations between the two classic arms of the immune system expands the paradigm of innate versus adaptive immunity and, thus, sheds doubt on the concept of “innate” versus “adaptive” immune-mediated diseases. The modern understanding of the immune system consists of an organizational continuum, rather than a dichotomic system, especially due to the acknowledgment of the bridge population subsets, which present functions comprehending the innate and adaptive poles (94). Macrophages, for example, can phagocyte and destroy microorganisms by the induction of oxidative burst, but bridges the gap between innate and adaptive immunity by processing and presenting antigens to lymphocytes. Other elements involved in the integration of innate and adaptive immunity include NKT cells, γδ T cells, CD8α T cells, and B1 cells. In addition, innate lymphoid cells also produce large amounts of cytokines attributed to adaptive immunity, such as IFNγ, IL-4 and IL-17A.

This review refers mainly to the innate arm of the immune system in BD. However, there are several pieces of evidence supporting the participation of the adaptive immunity in BD pathogenesis. Keller et al. (95) described a prominent CD4^+^CXCL8^+^CCR6^+^ T cell infiltrate in three different “neutrophilic” diseases: BD, pustular psoriasis and generalized exanthematous pustulosis. Interestingly, these cells produced predominantly CXCL8 and GM-CSF, but not IL-5 and IFNγ. Therefore, it is possible that these cells constitute a different subset of T cells, since their phenotype and functions differ from those of other classical CD4^+^ cells, such as Th1, Th2, Th17, and are associated to a unique inflammation cascade that promotes neutrophil hyperactivation.

Additionally, Th1 cells producing TNFα, IFNγ, IL-8, IL-12, CCR5, CXCR3, and MCP-1 (macrophage chemoattractant protein 1) were reported in several BD lesions, including oral and genital ulceration, pseudofolliculitis, pathergy pustules and bowel ulcers (88, 96). Data regarding regulatory T (Treg) cells are scarce and conflicting. Some studies demonstrated a high number of Treg cells (CD4^+^CD25^high^Foxp3^+^) in peripheral blood and cerebrospinal fluid (CSF) from BD patients (97–99). On the other hand, one study reported a decreased frequency of Treg in peripheral blood (100) and another one showed no difference in Treg frequency between BD and healthy controls. However, BD patients presented a decreased frequency of activated Treg cells (CD45RA^+^CD25^+++^) (101).

Th17 cells are also apparently important in BD pathogenesis, especially by recruiting neutrophils *via* G-CSF (102). Indeed, the percentage of peripheral Th17 cells and IL-17 production are increased in active BD (103). Noteworthy, T γδ and NKT cells are also capable of IL-17 production and apparently are associated with BD pathogenesis as well (104, 105). Geri et al. (101) demonstrated an increase in the number of Th17 cells and a reduction of Treg cells in the peripheral blood from BD patients, as well as increased serum levels of IL-21 compared to controls. In addition, healthy control CD4^+^ T cells stimulated *in vitro* with sera from active BD patients showed high IFNγ and IL-17A and decreased T reg cells differentiation compared to stimulus with sera from BD patients in remission. Moreover, Bassyouni et al. (106) showed that Th17 polarization in BD patients is induced by high levels of the inflammatory mediator serum amyloid-A. Thus, IL-17 axis seems to coordinate interactions between lymphocytes and neutrophils in BD and may represent a potential therapeutic target. In fact, the adaptive immune system apparently can stimulate neutrophil functions, contributing to the hyper-activated status of these cells. **Figure 1** summarizes a proposition for integrated pathogenesis of Behçet`s disease.

## Conclusion

Understanding Behçet’s disease pathogenesis is a pivotal step for the development of novel and efficacious therapies. Nevertheless, polygenic inheritance with the participation of several unknown environmental factors contributes to heterogeneity among patients and extra challenge for elucidating its pathogenesis. Evidence indicates that innate immunity is prominently involved in BD, which is illustrated by the striking neutrophil hyperactivity and its interaction with monocytes. However, adaptive immunity also seems to be important in BD, with particular emphasis on Th1 and Th17 responses.

### Key messages

1. Phagocyte hyperactivity, with increased oxidative burst and chemotaxis, is a hallmark of Behçet’s disease.
2. Soluble factors carried in the plasma contribute to phagocyte dysfunction in BD
3. Innate and adaptive immunity play an important role in BD pathogenesis and the IL-17 axis seems to play a pivotal role in the integration of the two arms of the immune system in this disease.

## Author Contributions

SP conceived, reviewed the literature, designed, wrote, and reviewed the manuscript. LA reviewed and improved the manuscript. AS reviewed and improved the manuscript. All authors contributed to the article and approved the submitted version.

## Funding

This study was supported by Fundação de Amparo à Pesquisa do Estado de São Paulo (FAPESP) n# 2011/50292-2 and by Research and Development from Fleury Group n# 12619. SP (grant #233205/2014-4) and LA (grant #310334/2019-5) are supported by the Brazilian research agency National Council for Research (CNPq).

## Conflict of Interest

The authors declare that the research was conducted in the absence of any commercial or financial relationships that could be construed as a potential conflict of interest.
